# Integration of EGA secure data access into Galaxy

**DOI:** 10.12688/f1000research.10221.1

**Published:** 2016-12-12

**Authors:** Youri Hoogstrate, Chao Zhang, Alexander Senf, Jochem Bijlard, Saskia Hiltemann, David van Enckevort, Susanna Repo, Jaap Heringa, Guido Jenster, Remond J.A. Fijneman, Jan-Willem Boiten, Gerrit A. Meijer, Andrew Stubbs, Jordi Rambla, Dylan Spalding, Sanne Abeln

**Affiliations:** 1 Department of Bioinformatics, ErasmusMC Rotterdam, Rotterdam, Netherlands; 2 Department of Computer Science, Vrije Universiteit, Amsterdam, Netherlands; 3 European Molecular Biology Laboratory, European Bioinformatics Institute, Wellcome Genome Campus, Hinxton, UK; 4 The Hyve, Utrecht, Netherlands; 5 Genomics Coordination Centre, UMC Groningen, Groningen, Netherlands; 6 ELIXIR Hub, Wellcome Genome Campus, Hinxton, UK; 7 Department of Urology, ErasmusMC Rotterdam, Rotterdam, Netherlands; 8 Diagnostic Oncology, Netherlands Cancer Institute, Amsterdam, Netherlands; 9 Lygature, Utrecht, Netherlands; 10 Centre for Genomic Regulation, Parc de Recerca Biomédica de Barcelona, Barcelona, Spain

**Keywords:** Galaxy, EGA, bioinformatics, workflows, translational research, data management

## Abstract

High-throughput molecular profiling techniques are routinely generating vast amounts of data for translational medicine studies. Secure access controlled systems are needed to manage, store, transfer and distribute these data due to its personally identifiable nature. The European Genome-phenome Archive (EGA) was created to facilitate access and management to long-term archival of bio-molecular data. Each data provider is responsible for ensuring a Data Access Committee is in place to grant access to data stored in the EGA. Moreover, the transfer of data during upload and download is encrypted. ELIXIR, a European research infrastructure for life-science data, initiated a project (2016 Human Data Implementation Study) to understand and document the ELIXIR requirements for secure management of controlled-access data. As part of this project, a full ecosystem was designed to connect archived raw experimental molecular profiling data with interpreted data and the computational workflows, using the CTMM Translational Research IT (CTMM-TraIT) infrastructure http://www.ctmm-trait.nl as an example. Here we present the first outcomes of this project, a framework to enable the download of EGA data to a Galaxy server in a secure way. Galaxy provides an intuitive user interface for molecular biologists and bioinformaticians to run and design data analysis workflows. More specifically, we developed a tool -- ega_download_streamer - that can download data securely from EGA into a Galaxy server, which can subsequently be further processed. This tool will allow a user within the browser to run an entire analysis containing sensitive data from EGA, and to make this analysis available for other researchers in a reproducible manner, as shown with a proof of concept study.  The tool ega_download_streamer is available in the Galaxy tool shed: https://toolshed.g2.bx.psu.edu/view/yhoogstrate/ega_download_streamer.

## Introduction

**Figure 1.**
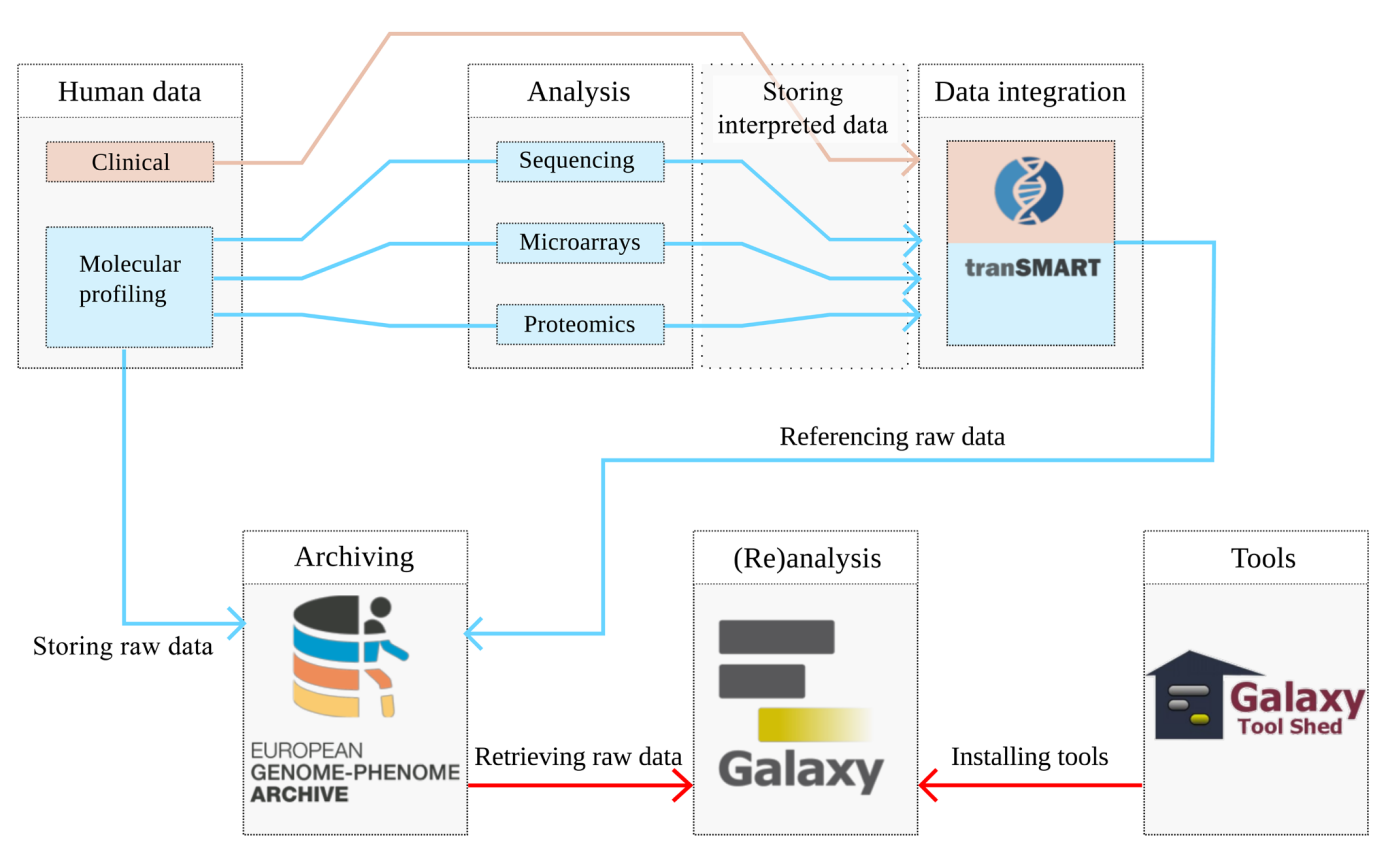
A flowchart of the designed ecosystem for the management and storage of data for clinical research data with a focus on security. The clinical data of an experiment describes the clinical-pathological data, including tissue and patient information. Descriptors of the samples combined with these variables are stored in tranSMART. Molecular profiling data are derived from samples of patients: these samples are processed in the laboratory to obtain tissue derivatives, such as isolation of DNA, RNA and proteins, which are subsequently analysed by high throughput experimental techniques to obtain the raw molecular profiling data; the descriptions of the performed experiments are also stored in tranSMART. The actual raw data produced by the high throughput analysis are physically stored in repositories like EGA, while the interpreted data processed by extensive computational workflows, and references to the raw data are stored in tranSMART. The ability to reanalyse the raw data, is provided by Galaxy. Note that the work described here indicated by red arrows implements a data connection, allowing a user to retrieve raw data from EGA in Galaxy, and run subsequent workflows, constructed by tools in the Galaxy tool shed.

With the advent of high-resolution and high-throughput experimental platforms the field of biomedical research has become more complex, with major shifts in data diversity and dimensions. Consequently, solutions for the increasing demand of data processing, storage and workflow management are required for translational research. Due to the privacy issues related to the clinical nature of translational research and personal footprints in molecular data, there is a need for a secure framework to store and analyse data. The aim of CTMM Translational Research IT (CTMM-TraIT) project is to provide a multidomain IT-infrastructure as an end-to-end solution where researchers can capture, process, and share their study data. To achieve this, CTMM-TraIT makes use of large community-driven open source software including tranSMART ^1– 3^ and Galaxy ^4, 5^. In a collaboration between ELIXIR, CTMM-TraIT and European Genome-phenome Archive(EGA) a full ecosystem was designed, as shown in Figure 1, to connect the storage of raw molecular profiling data with processed data and the computational workflows.

### Storage

Facilitating the long-term storage and management of raw, interpreted and clinical data (patient and tissue information), supported by provenance of computational workflows, is a key aim of the CTMM-TraIT project; special attention to the security is necessary, due to the privacy-sensitive nature of the data. EGA is a service that provides long term archiving and distribution of identifiable genetic and phenotypic data resulting from biomedical research projects. Data stored at the EGA are collected from individuals whose consent agreements authorise release only for specific research use to bona fide researchers. Strict protocols govern how information and data are managed, stored, transferred and distributed by the EGA project, and each data provider is responsible for ensuring a Data Access Committee is in place to grant access to their data. However, EGA only functions as long-term storage facility and does not facilitate analysis. Within the CTMM-TraIT project, we agreed upon a workflow in which the interpreted data, such as the BAM files, and the clinical-pathological data would be stored in tranSMART; the raw and uninterpreted data, such as FASTQ and BAM files, would be stored and archived in EGA. Figure 1 demonstrates how the clinical-pathological and interpreted data are managed by tranSMART, which links to the raw data in EGA which in turn can be accessed and (re)analysed from within a Galaxy environment.

### Integrated analysis

Within EGA, data are separated into different layers: 1) raw data, produced by high-throughput platforms; 2) metadata describing the raw data, e.g. machines and protocols used and descriptions of treatments and tissues and 3) interpreted data, produced by running analyses on the first two layers. Since the EGA is ideally placed to facilitate continued data access and management to funded projects after completion of the project, and the data from layer 3 should be reproducible using the data from the other layers, only the data from the 1st and 2nd layer will go to EGA for archival storage.

In the ecosystem we use Galaxy, a popular and user friendly web-based bioinformatics platform that provides an intuitive user interface to run and design workflows, to perform integrated analysis from multiple domains (genomics, transcriptomics and proteomics), and to share and communicate both results and methodologies. It makes use of tools and libraries provided by the bioinformatics community as plugins. Tools are embedded as plugins in such a way that each of them becomes a modular block that can be plugged into the next block (tool or visualisation) ^6^. To directly import and hence analyse data stored in EGA within Galaxy, it is necessary to implement an interface from EGA to Galaxy as a plugin (Galaxy tool wrapper).

Here we present an end-to-end interface to a framework which seeks to extend data accessibility, ensures long-term archival and facilitates downstream analysis by utilising EGA. The framework embeds EGA access into Galaxy, and allows subsequent workflows using (novel) Galaxy tools. An advantage of setting up an analysis in this way is that both the tools and the data are connected and centralised and can be shown, shared, and reproduced. We further demonstrate the setup with an RNA-Seq use case.

## Methods

### Implementation

We have embedded the EGA download client ( https://ega-archive.org/using-ega-download-client) into Galaxy as a tool wrapper including dependency management. The tool is named as ega_download_streamer and can be installed on Galaxy systems from the main tool shed. Before being able to use the tool, Galaxy needs to be configured by setting up an EGA account, further explained in Supplementary File 1. Hereafter, we call this tool "Galaxy EGA download streamer"

To allow access to EGA, the tool interfaces with the EGA download client ensuring that encrypted data are transferred from EGA. The Galaxy EGA download streamer gets data from EGA directly into the user’s history. On the Galaxy side, the server starts with a form requesting a unique EGA file identifier. After submission, it logs in with the configured credentials, creates an encryption key and sends this over a secure connection to EGA requesting EGA to encrypt the file that corresponds to the identifier with the given key. After making the request, the encrypted package becomes available and will be downloaded; subsequently the connection with EGA is closed. The package will be locally decrypted and extracted if it is a file archive. Galaxy determines, with its built-in sniffing system, the file type (FASTQ, BAM, GTF, etc.) and eventually puts the files into the user’s history.

### Operation

Galaxy with a version number 16.07 and above is required to use this tool, because only this version and higher can make the tool detect data types automatically within a workflow. In addition, at least 30 GB RAM and 100GB hard disk space is required to run the use case in the next section. Other system requirements for the installation of Galaxy can be found in Galaxy official document ( https://wiki.galaxyproject.org/Admin/GetGalaxy).

## Use case

**Figure 2.**
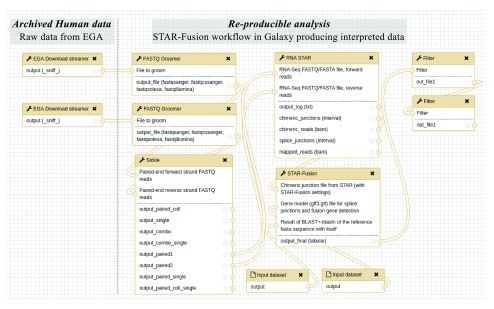
STAR-Fusion workflow in Galaxy. The workflow firstly, obtains the raw data from EGA, to subsequently allow reanalysis of the data in a workflow of multiple components, to derive interpreted data. The raw forward and backward FASTQ sequencing reads are imported from EGA by ega_download_streamer; subsequently, the tool FASTQ Groomer does a consistency check of the data formats; then with Sickle, low quality bases (Q<30) are trimmed and reads clipped into less than 25 bases are discarded, only outputting the high-quality sequencing reads. Afterwards, these reads are aligned to the hg19 (GenBank Assembly ID GCA_000001405.1) reference genome in RNA STAR. Then STAR-Fusion is used for predicting the fusion genes, which also requires two reference files as auxiliary inputs. The output goes through two filters to only keep predictions having more than two split reads and more than two spanning reads.

As a proof of concept we show how the Galaxy EGA download streamer may be used in a workflow to detect fusion genes from RNA-seq data. To demonstrate this workflow we use cell line data that can be made publicly available. We use an RNA-Seq dataset of the VCaP cell line in the Galaxy workflow shown in Figure 2. Since the recurrent fusion TMPRSS2-ERG is found in more than 50% of the diagnosed patients ^7^, we test it for the presence of fusion genes, and since VCaP contains TMPRSS2-ERG ^8^ we can use TMPRSS2-ERG as a positive control. We use the tool STAR-Fusion ( https://github.com/STAR-Fusion/STAR-Fusion/wiki) which can be used as a separate module after running the RNA-STAR aligner ^9^.

Besides the Galaxy EGA download streamer accessing EGA, this workflow also requires adaptations to the RNA-STAR Galaxy wrapper from the IUC group ( https://toolshed.g2.bx.psu.edu/repos/iuc/rgrnastar), by adding a specific fusion detection settings preset and to create a new Galaxy wrapper for STAR-Fusion. The workflow starts with obtaining data from EGA which for this study are the raw paired-end FASTQ sequencing reads. These files translate to EGA identifiers: EGAF00001210838 (forward) and EGAF00001210839 (reverse) and will be the input for the Galaxy EGA download streamer. Because we want to ensure the handshake with other tools and the several sub file formats of FASTQ ^10^, it is desirable to proceed with a FASTQ-sanger encoded file, which is ensured by the tool FASTQ Groomer ^11^. Note that the search space for alignment is higher for fusion gene detection than for most other alignment purposes such as determining expression levels; hence we would like to have a high base quality to avoid misalignments and unnecessary computation. We improve the base quality by trimming low quality bases (Q<30) and discarding reads being clipped into less than 25 bases with the tool Sickle ( 12; https://github.com/najoshi/sickle). These high-quality sequencing reads were aligned to the hg19 (GenBank Assembly ID: GCA_000001405.1) reference genome. As proposed by the authors of STAR-Fusion we use fusion gene detection specific settings available as the “Use parameters suggested for STAR-Fusion”-preset in IUC’s Galaxy RNA-STAR wrapper. Besides a classical alignment file, it also produces an alignment file for the discordant reads and an equivalent junction file. STAR-Fusion uses the junction file to predict fusion genes and requires two additional reference files (Data and software availability). STAR-Fusion produces a list that contains many candidates including predictions that have a rather low confidence level, less than 3 split or spanning reads. Therefore we end the workflow with two filters that only keep predictions that have more than two split reads and more than two spanning reads.

The results on the VCaP data contain candidate fusion genes with a high number of corresponding reads: HNRNPC-KIAA0586, USP10-ZDHHC7, TMPRSS2-ERG, PIK3C2A-TEAD1. Except for HNRNPC-KIAA0586, the others were earlier reported in RNA-Seq and DNA-Seq analysis ^11, 13, 14^.

## Discussion and conclusions

The EGA-TraIT implementation study sets out to design an entire ecosystem for molecular profiling data in clinical research, with a focus on security. Here we demonstrate with a proof-of-concept study that it is possible to connect EGA and Galaxy as designed within this system. This study is part of an ongoing effort to make EGA data correspond to FAIR (Findable, Accessible, Interoperable, and Reusable) data principles ^15^, which will result in further recommendations on the EGA data model and ontologies in the near future. Here we highlight the implementation of the storage component and demonstrate how to use it in an analysis context. Its key value is that it allows tracking and redistributing the entire workflow and data jointly, from the beginning to the end, ensuring the provenance of all intermediate layers up to the final results. As a result, we have:

- shared molecular data via EGA;
- created new Galaxy tools;
- shared the workflow, including all parameters, via a URL for Galaxy (as a shared history) and via myExperiment ^16^;
- shared the interpreted data as a Galaxy history;
- shared a manual as a Galaxy page on how to set up such an experiment.

Further to the work described here, the implementation study continues until the end of 2016 and the complete outcomes from this project, with recommendations on structuring metadata, will be presented in a future report. This implementation study and the IMI OncoTrack implementation study ( https://www.elixireurope.org/news/elixir-and-oncotrack-examine-solutions-long-term-management-translational-data), have provided complementary use cases for EGA to shape linkage with external databases, such as tranSMART.

### Recommendations

A limitation of the working prototype for the Galaxy EGA download streamer is that it requires setting up a generic EGA account for the entire public Galaxy server. This means that any user can only access the data files that are available for that generic account rather than a personal account. We have thought of several solutions:

- A secure input type for passwords. However, Galaxy currently does not support password input types, and textual input types are recorded in the database which allows them to be shared when history items are shared with other users.
- Adapt EGA in a way that it shares tokens that allow download of a particular file within a particular time. However, re-running the tool would require selecting a new token. For this setup, it would be ideal to have a non-memorised data type.
- An authentication management mechanism within Galaxy. If a user would configure certain authentications within Galaxy, Galaxy can manage these authentications and automatically connect to EGA on request (OAuth model).

Due to the current limitation of data protection and access control in a public Galaxy service, a private Galaxy instance seems to be a practical solution to this problem, keeping the data access limited to a small research group. This does require extra expertise to properly establish the service in a secure environment.

## Data and software availability

### Tools

The software can be found in the main Galaxy tool shed at the following URL: https://toolshed.g2.bx.psu.edu/view/yhoogstrate/ega_download_streamer

The source code of Galaxy EGA download streamer is available at Github: https://github.com/ErasmusMC-Bioinformatics/galaxytools-emc

Archived source code of Galaxy EGA download streamer at the time of publication: https://zenodo.org/record/167330; doi, 10.5281/zenodo.167330 ^17^ License: MIT

### Workflow

The workflow is publicly accessible and can be downloaded at: https://bioinf-galaxian.erasmusmc.nl/galaxy/u/yhoogstrate/w/ega-vcap-rna-seq-demo

The workflow with corresponding data are explained in more detail at the published Galaxy page, including a description of the results: https://bioinf-galaxian.erasmusmc.nl/galaxy/u/yhoogstrate/p/egavcap-rna-seq-star-fusion-demo

The workflow is also described at myExperiment: http://www.myexperiment.org/workflows/4924.html

The data are accessible at the following URL: http://bioinf-galaxian.erasmusmc.nl/galaxy/u/yhoogstrate/h/galaxy-ega-star-fusion-demo

### Data

The raw paired-end FASTQ sequencing reads used in the "Use case" section can be downloaded from EGA using EGA File identifiers: EGAF00001210838 (forward) and EGAF00001210839 (reverse). There files belong to EGA dataset EGAD00001001626, which belongs to the study that can be accessed from https://ega-archive.org/studies/EGAS00001001476.

Two additional reference files required in the workflow can be downloaded from the "Shared Data" section of Galaxy: https://bioinf-galaxian.erasmusmc.nl/galaxy/library/list# folders/F8f0c64b106db6693

## Supplementary material

**Supplementary File 1:** A user guide for Galaxy EGA download streamer, including installation and usage.
