# Supplementary material for: Integration of EGA secure data access into Galaxy
Source: F1000Res. 2016 Dec 12;5:ELIXIR-2841. [Version 1] doi: 10.12688/f1000research.10221.1 (PMC5302147; doi:10.12688/f1000research.10221.1)
Supplement: Supplementary file 1 [file f1000research-5-11010-s0000.tgz › 5bd3a2ed-6b9c-468a-946c-578a836d04db.pdf]

A User Guide for Galaxy EGA Download Streamer

November 24, 2016

This document is serving as the supplementary material for the paper "Integration of EGA Secure Data Access into Galaxy".

Installation

The Galaxy EGA download streamer is available in the public tool sheds under the name `ega_download_streamer` and can be viewed at the following URL: `https://toolshed.g2.bx.psu.edu/view/yhoogstrate/ega_download_streamer`

The Galaxy web administrator can install this tool in the admin menu, under the tabs "Tools and Tool Shed" then "Search Tool Shed". In here, the admin needs to go to the Galaxy main tool shed where the search term `ega_download_streamer` should show the tool installer maintained by *yhoogstrate*. By pressing it, a new page will open. This page will indicate that it will automatically install the tool dependencies and shows the README, including the LICENSE, and a button that will install the entire EGA software package as dependency into the Galaxy server.

Because Galaxy has not yet supported a password input type, the Galaxy administrator has to configure Galaxy to set up one generic EGA account. This is done by adding lines, setting up the proper environment variables, to a destination (computer node) in Galaxy's `./config/job_conf.xml`:

```
<destination id="local_dest" runner="local">
    <env id="user">email</env>
    <env id="pass">password</env>
</destination>
```

The Galaxy EGA download streamer is written in such a way that these credentials will not be exposed, e.g. when sharing history items in Galaxy. The `runner="local"` must correspond to the runner that runs the `ega_download_streamer` (only for advanced setups; local is default).

Bugs and other contributions to the Galaxy EGA download streamer are more than welcome at: `https://github.com/ErasmusMC-Bioinformatics/galaxytools-emc`

Usage

To use this Galaxy EGA download streamer in a properly configured Galaxy server, the user should select the tool "EGA Download streamer" in the tool menu (Figure 1):

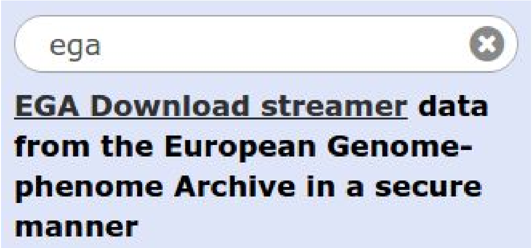

Figure 1: "EGA Download streamer" in the tool menu

After clicking the tool, a new page is rendered, where an EGA file identifier should be filled in (Figure 2). An EGA file identifier can uniquely identify one file in EGA archive.

The download starts after the execute button is clicked. It usually takes a while to get the files downloaded, which is dependent on the size of the files and the network connections. Subsequently, the data downloaded from EGA may be used in a user-built or existing Galaxy workflow, as shown in the section "Use case" of this paper.

The workflow and auxiliary reference files can be downloaded and imported into Galaxy where the workflow is run to reproduce the results in this paper.

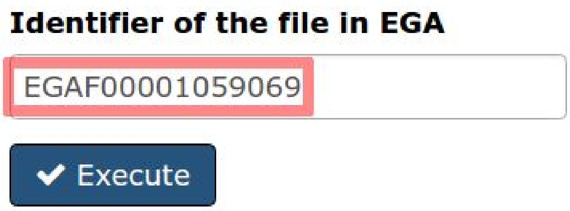

Figure 2: The Galaxy EGA download streamer takes an EGA file identifier as input. In EGA, a file identifier always begins with "EGAF", uniquely identifying one file in EGA archive. With proper access rights, the raw data can be fetched from EGA by this identifier.
