# Supplementary material for: Integration of EGA secure data access into Galaxy
Source: F1000Res. 2016 Dec 12;5:ELIXIR-2841. [Version 1] doi: 10.12688/f1000research.10221.1 (PMC5302147; doi:10.12688/f1000research.10221.1)
Supplement: Peer review history

# Peer review: Referee response for version 1

**DOI:** 10.5256/f1000research.11010.r19969

**Published:** 2017-02-06

**Authors:** Anton Nekrutenko

**Affiliations:** 1 Department of Biochemistry and Molecular Biology, Penn State University, University Park, Pennsylvania, PA, USA

This manuscript describes proof of principle for accessing the data contained within the European genome-phenome archive (EGA) via Galaxy analysis environment. This is a very timely and important development as it would dramatically increase the utility of resources such as EGA. This is because continuous accumulation of new data and development of new technologies will undoubtedly result in the need to reanalyze previously generated datasets and to combine them with newly acquired research outcomes. Having ability to securely request and analyze the data via Galaxy makes such re-analyses straightforward and convenient resulting in more researchers performing these tasks. My hope is that this would will push other sites with similar data (e.g., dbGaP) to implement similar software solutions.

 I have several comments:

 To access the data a user must have necessary security credentials. One the data is transferred into Galaxy it leaves the EGA filesystem. How do authors secure this particular Galaxy instance? Other sites housing protected data will need this information to make the decision on whether to adopt a similar strategy.

 It is necessary to explain why the Galaxy instance for EGA access requires a minimum of 30 GB RAM and 100GB disk space. It seems that these requirements are dictated by data size and types of tools (i.e., STAR tools require considerable amount of memory to perform analyses of RNAseq data). Again other sites adopting Hoogstrate’s *et al*. approach should be aware of the fact that Galaxy itself requires little resources. Instead the underlying tools and data would dictate hardware specifications.

 Galaxy Toolshed is introduced in the paper with little explanation of what it is. A couple of sentences descripting this “AppStore” will be helpful to reads who are not familiar with Galaxy’s ecosystem.

 While it is a good starting example, it would be helpful to show that one can generate more concrete results with Galaxy beyond just filtering STAR-fusion output. Is there any interesting bit of biological information that can be added to the paper?

 When I tried to access a history at https://bioinf-galaxian.erasmusmc.nl/galaxy/u/yhoogstrate/w/ega-vcap-rna-seq-demo browser has timed out.

I have read this submission. I believe that I have an appropriate level of expertise to confirm that it is of an acceptable scientific standard.

# Peer review: Referee response for version 1

**DOI:** 10.5256/f1000research.11010.r19733

**Published:** 2017-02-02

**Authors:** Hervé Ménager

**Affiliations:** 1 Center of Bioinformatics, Biostatistics and Integrative Biology, Pasteur Institute, Paris, France

Hoogstrate & al. present a Galaxy plugin (tool) to enable a direct access to the European Genome-phenome Archive. The purpose of this work is to enable the direct access from the Galaxy workbench to the data stored in the EGA, whose access is restricted due to their personal nature (personally identifiable biomedical data). Such components are indeed a requirement to implement a user-level access to restricted data, and are aligned with the goals of CTMM-TraIT and ELIXIR. The proposed solution is simple, pragmatic and effective.

 The current limitation of this work is the authentication for users who have to share their credentials at the level of the Galaxy instance. It is very clearly explained, and I agree that this restricts the usage of this tool to small group or private instances. This very problem is however discussed by the Galaxy community (see https://github.com/galaxyproject/galaxy/pull/393, https://github.com/galaxyproject/galaxy/pull/3121, https://github.com/galaxyproject/galaxy/pull/3383) and hopefully solving it will enable such use cases on institution-wide and public Galaxy servers.

 One aspect I would like to see discussed, although it is probably a bit beyond the scope of this paper, is the implementation of the first analysis step (figure 1) of the designed ecosystem which produces the interpreted data stored into TransMart. Ideally, if the Galaxy system is used for reanalysis of the raw and interpreted data, one would expect that the "Analysis" use the same Galaxy workflows. It does not seem to be the case here, and I would be very interested in hearing what motivates selecting the alternative that was preferred to this solution.

I have read this submission. I believe that I have an appropriate level of expertise to confirm that it is of an acceptable scientific standard.
